# Occupational exposures and risk of pulmonary alveolar proteinosis (PAP)

**DOI:** 10.5271/sjweh.4275

**Published:** 2026-05-01

**Authors:** Kjell Torén, Anna-Carin Olin, Maria Åberg, Kristin J Cummings, Linus Schiöler, Paul D Blanc

**Affiliations:** 1 Occupational and Environmental Medicine, School of Public Health and Community Medicine, Sahlgrenska Academy, University of Gothenburg, Gothenburg, Sweden.; 2 Department of Occupational and Environmental Medicine, Sahlgrenska University Hospital, Gothenburg, Sweden.; 3 General Practice/Family Medicine, School of Public Health and Community Medicine, Institute of Medicine, Sahlgrenska Academy, University of Gothenburg, Gothenburg, Sweden.; 4 Regionhälsan, Västra Götalandsregionen, Gothenburg, Sweden.; 5 Occupational Health Branch, California Department of Public Health, Richmond, CA, USA.; 6 Division of Occupational, Environmental and Climate Medicine, Department of Medicine, University of California, San Francisco, CA, USA.

**Keywords:** case–control study, epidemiology, lung disease, organic dust

## Abstract

**Objectives:**

Occupational exposures to dust have been associated with pulmonary alveolar proteinosis (PAP) in case series, but population-based epidemiological data are needed.

**Methods:**

We identified 286 cases of PAP from the Swedish National Patient Register and the Cause-of-Death Register between 1991 and 2022. For the present analysis, we included 212 cases aged 20–65 years with available occupational information before the index date or within two years thereafter. Controls matched on age and sex were drawn from the population register and assigned the same index date as their corresponding case; of these, 1438 controls had available occupational information and were included in the analyses. We linked cases and controls to Swedish registries to obtain socioeconomic status and occupational data. We applied an established job-exposure matrix to characterize occupational exposure to inorganic dust, with the subset silica dust, fumes, vapors and gases and organic dust. We used adjusted conditional logistic analyses to estimate the odds ratios (OR) with 95% confidence intervals (CI) for the occupational exposures in the year before index date and PAP.

**Results:**

None of the occupational exposures analyzed showed a statistically significant association to PAP. The OR for inorganic dust was 1.08 (95% CI 0.75–1.55); silica dust alone was 1.55 (95% CI 0.75–3.23) and organic dust was 1.48 (95% CI 0.92–2.38). Among men, however, exposure to organic dust was associated with PAP [OR 1.92 (95% CI 1.18–3.23)]. Among women, the results were inconclusive.

**Conclusions:**

There were no associations between occupational exposure to fumes, vapors and gases and inorganic dust and risk of PAP. Among men, exposure to organic dust was associated with increased risk for PAP. Some occupational inhalants may increase the risk of PAP.

Pulmonary alveolar proteinosis (PAP) was first described as such in 1958 (1). That initial publication reported 27 cases, of which 13 had occupations implicating occupational exposure to dust, 9 to organic dust, and 3 to silica. A rare and sometimes fatal disease, PAP is characterized by progressive accumulation of surfactant lipids and proteins in the lungs. The disease usually manifests in adults as progressive breathlessness, fatigue and impaired exercise capacity over a longer period. There are three subtypes currently described: hereditary, autoimmune, and secondary, although these groupings overlap (2, 3). Of note, the different forms of PAP are all characterized by similar pathology suggesting a final common mechanistic pathway (4). In PAP, especially the autoimmune form, the function of granulocyte/macrophage colony stimulating factor (GM-CSF) is affected. GM-CSF is critically important for alveolar macrophage function and its ability to clear surfactant (3). This disturbed function is believed to cause massive accumulation of surfactant lipids and debris in the alveolar space, leading to the progressive respiratory insufficiency characteristic of PAP (5). The annual worldwide incidence of PAP is estimated to be approximately 2 cases/million persons per year (6–8). Peak incidence is observed at 30–40 years of age. Men have higher incidence than women, which could suggest an occupational contribution to the disease. Tobacco smoking has not been shown to be an established risk factor for PAP onset, although current smoking seems to be common in some case series, further supporting the importance of noxious exposures (6, 9). Tobacco smoking has also been associated with more severe disease (10). The epidemiology of PAP has been based largely on clinical case series.

Importantly, a consistently large component of PAP case series has been characterized by associated occupational exposures, most notably organic dust and silica. A review, which pooled data for 1539 published cases of PAP, estimated that the work-related attributable fraction for this disease was 29% [95% confidence interval (CI) 21–37%] (11). The reported occupational exposures were inorganic dust, especially silica dust. Another review of PAP concluded that a substantial fraction is associated with occupational inhalational exposures, mainly inorganic dust, silica and different organic dusts, such as flour and wood (12).

The lack of population-based epidemiological studies of occupational risk factors for PAP represents a critical research gap. Recognizing how rare this condition is, the paucity of such epidemiology investigations is not surprising, given that such investigations would require a large national or even multinational study. Importantly, Sweden provides access to comprehensive national patient and mortality registries, as well as annual updating of occupational status and other socioeconomic factors. We have leveraged this by conducting a robust case–control study investigating occupational risk factors for PAP to quantify their contribution for the overall burden of PAP.

## Methods

### Establishment of the study population

We selected from the Swedish Mortality Register and the Swedish National Inpatient Register for 1991–2022 all cases of PAP defined according to International Classifications of Diseases (ICD) as J84.0 (ICD 10), and 516A (ICD 9) in the age interval 20–65 years. We assigned each case an index date: the first date the disease was registered in either the National Inpatient Register or the Mortality Register. The National Inpatient register includes all hospital-based events as well as hospital-associated outpatient polyclinical visits. We selected six living controls for each case, matched for gender and age (case calendar-year of birth) by index date from the Swedish Historical National Population Registry. The controls were assigned the same index date as the corresponding case.

We linked cases and controls with LISA, the Swedish national longitudinal integration database for health insurance and labor market studies, to extract the highest educational level attained, categorized as: pre-highschool (up to 9 years); completed highschool; or university examination. We also extracted from LISA a complete occupational history from 1990, 1995, and annually from 2001.

### Classification of occupational exposures

All occupations among cases and controls in 1990, 1995 and from 2001 were classified by four-digit level code according to International Standard Classification of Occupations (ISCO) (13, 14). To categorize exposures, we applied a previously published job-exposure matrix (JEM) for inorganic dust including silica dust; silica dust alone; fumes; vapors and gases; and organic dusts (15). The JEM-defined levels were further classified as unexposed, low, or high, with low and high levels also merged as alternative categorization of “all exposed” (15). Fumes were defined as smoke from various combustion processes, such as from welding, fires, and from second-hand tobacco smoke. Vapors and gases were defined as substances in aerosol or gas phase. Inorganic dust including silica dust mainly comprised exposure to mineral dust, quartz, metals and concrete dust. We also defined exposure to inorganic dust without silica exposure and silica exposure only. Organic dust included wood dust, agricultural dust, and flour and grain dust. Study participants could be classified as exposed to several agents.

### Statistical methods

We used conditional logistic regression to estimate the association of PAP and JEM-defined exposures expressed as odds ratios (OR) and their 95% CI. The exposures were either classified in three groups – unexposed, low exposure, or high exposure – or alternatively in two groups as unexposed versus low or high exposure combined (all exposed). The intention of the main analyses was to test the association of occupational exposure in the year before disease onset. In cases that lacked an occupation the year before the index date, we selected the occupation closest to the index date, with a maximum of seven years before the index date or two years after the index date. The basic regression model (Model 1) was adjusted only for matching strata (eg, equivalent to adjusting for gender, age, and index date). The adjusted model (Model 2) included, in addition, educational levels. We tested each of the JEM-defined categories of exposures in separate models and also performed an analysis including all occupational exposure groupings in the same model. One model included all main exposures, which we additionally stratified among men and women separately. A second set of analyses included all the different exposures but with inorganic dust separated into inorganic dust without silica and silica exposure only. In that model, women were excluded due to too few cases in selected cells.

Furthermore, we analyzed the effects of the cumulative exposure (five years) for the 148 cases and their controls where we had occupational information for five years preceding the index date, defined as the number of years exposed (ie, 0–5). For this analysis we used multivariable logistic regression adjusted for gender, age, index date, and educational level.

We also analyzed the effects of the cumulative exposure (five years) for the 148 cases and their controls where we had occupational information for five years preceding the index date, defined as the number of years exposed (ie, 0–5). The cumulative exposure was treated as a continuous variable assuming a linear relation to the log odds of the outcome. Since the assumption of a linear, unit increment by year may or may not be correct, we approached this analysis as a test of linear trend, eg, a test of the null hypothesis of no association per year of exposure. Hence, no OR is presented based on this model. For this analysis, we used multivariable logistic regression adjusted for gender, age, index date, and educational level.

All statistical analyses were performed using the SAS version 9.4 M7 software (SAS Inc. Cary, NC, USA). P-values for the trend were calculated by treating the exposures as continuous variables.

## Results

We identified 286 cases of PAP over the period 1991 through 2022. Of those, 272 originated from the National Patient Registry and 14 from the Cause-of-Death Register. We randomly selected 1716 controls, 6 for each case. Of the cases and controls, we identified any occupation before the index date or two years after in 212 cases (74.1%) and 1438 controls (83.8%). There were 148 cases with an occupation the exact year before index date and, for 64 cases, we used the occupation at a maximum of seven years before or two years after the index date. For analyses involving occupational exposures, inclusion was restricted to cases and controls with identifiable occupational information recorded before the index date or up to two years thereafter. Of the 212 cases, 204 originated form the National Patient Registry and 8 from the Cause-of-Death Register. In one case, a man exposed to inorganic dust lacked educational information so the case was excluded from the education-adjusted models (211 rather than 212 cases). Of the 212 included cases, 16 (7.5%) were from the period 1991–2000, 89 (42%) from 2001–2010, and the majority, 107 (50.5%) from the period 2011–2022. Descriptive data for the final study group, cases and controls, including their occupational data are shown in table 1.

**Table 1:** Characteristics of cases with pulmonary alveolar proteinosis (PAP) and matched controls from the general population of Sweden including occupational exposures defined by a job-exposure matrix. [IQR=interquartile range.]

		All PAP (N=212)			Controls (N=1438)	
		N (%)	Median (IQR)		N (%)	Median (IQR)
Males		103 (48.6)			701 (48.7)	
Age (years)			52.0 (42.0–60.0)			53.0 (43.0–61.0)
Completed university exam		66 (31.3)			510 (35.6)	
Occupational exposure						
	Inorganic dust	46 (21.7)			278 (19.3)	
	Silica dust	10 (4.7)			43 (3.0)	
	Vapors and gases	43 (20.3)			301 (20.9)	
	Organic dust	25 (11.8)			120 (8.3)	
	Fumes	35 (16.5)			281 (19.5)	

There were no statistically significantly increased odds for PAP for any of the JEM-defined exposure categories (table 2). Of note, however, the point estimates for silica dust and organic dust were OR 1.55 (95% CI 0.75–3.23) and 1.46 (95% CI 0.91–2.35), respectively. When considering low and high exposure, there was a step-up for organic dust (P=0.09) from OR 1.39 (95% CI 0.85–2.28) to 2.82 (95% CI 0.52–15.42) (table 2). There was also a step-up (P=0.11) for silica dust from OR 1.26 (95% CI 0.54–2.91) to 3.96 (95% CI 0.87–17.97). Of note, the number of cases were very low, two and three respectively, in the highly exposed groups. The estimates were similar, but somewhat lower, from the model with additional adjustment for educational level, as shown in table 2.

**Table 2:** Logistic regression models of risks of pulmonary alveolar proteinosis (PAP) (N=212) in relation to occupational exposure to inorganic dust, silica dust, vapor and gases, fumes, and organic dust. All exposed, low exposed and high exposed are compared with unexposed. [OR=odds ratios; CI=confidence interval; NA=not available].

Occupational exposures		Basic model ^a, b^			Adjusted model ^c^	
		OR	95% CI		OR	95% CI
All						
Inorganic dust including silica						
	All exposed (N=46)	1.08	0.75–1.55		1.00	0.68–1.47
	Low (N=43)	1.02	0.70–1.49		0.95	0.65–1.41
	High (N=3)	3.88	0.86–17.56		3.62	0.80–16.47
Silica dust						
	All exposed (N=10)	1.55	0.75–3.23		1.53	0.73–3.18
	Low (N=7)	1.26	0.54–2.91 ^d^		1.25	0.54–2.88 ^e^
	High (N=3)	3.96	0.87–17.97 ^d^		3.77	0.83–17.15 ^e^
Vapor and gases						
	All exposed (N=43)	0.91	0.64–1.31		0.89	0.62–1.28
	Low (N=7)	0.96	0.67–1.38		0.94	0.65–1.35
	High (N=0)	N.a.	N.a.		N.a.	N.a.
Organic dust						
	All exposed (N=25)	1.46	0.91–2.35		1.38	0.85–2.24
	Low (N=23)	1.39	0.85–2.28 ^f^		1.31	0.79–2.18 ^g^
	High (N=2)	2.82.	0.52–15.42 ^f^		2.71	0.49–14.84 ^g^
Fumes						
	All exposed (N=35)	0.75	0.51–1.11		0.72	0.49–1.08
	Low (N=35)	1.39	0.85–2.28 ^f^		1.31	0.79–2.18 ^g^
	High (N=0)	NA	NA		NA	NA

^a^ Matched for gender and age. ^b^ N=212. ^c^ Adjusted for educational level (N=211). ^d^ P for trend=0.11. ^e^ P for trend=0.13. ^f^ P for trend=0.09. ^g^ P for trend=0.14.

Amongst men, there was a statistically significant association for exposure to organic dust, OR 1.92 (95% CI 1.16–3.17), which was slightly lower after adjusting for educational level (table 3). When considering low and high exposure separately for organic dust amongst men, there was a step-up (P=0.006) from OR 1.78 (95% CI 1.06–3.01) to 5.73 (95% CI 0.81–40.76). For inorganic dust, silica dust, and vapor and gases there were no increased risk estimates; only for fumes the point estimate of the OR was <1.0. Of note, for inorganic dust and silica dust manifested the highest point estimates for the OR, but with CI including unity (table 3). Amongst women, there were no increased risk estimates (table 3).

**Table 3:** Logistic regression models of risks of pulmonary alveolar proteinosis (PAP) among men and women in relation to occupational exposure to inorganic dust, silica dust, vapor and gases, fumes, and organic dust during the year preceding the index date (onset of PAP). All exposed, low exposed and high exposed are compared with unexposed. [OR=odds ratios; CI=confidence interval; NA=not available].

Occupational exposures		Basic model ^a^		Adjusted model ^b^
		OR (95% CI)		OR (95% CI)
**Men (N=103)**				
Inorganic dust including silica				
	All exposed (N=34)	1.21 (0.77–1.89)		1.05 (0.66–1.67)
	Low (N= 31)	1.13 (0.71–1.79)		0.98 (0.60–1.58)
	High (N=3)	3.97 (0.88–18.00)		3.39 (0.74–15.53)
Silica dust				
	All exposed (N=8)	1.44 (0.64–3.24)		1.37 (0.61–3.09)
	Low (N=5)	1.08 (0.41–2.84)		1.04 (0.39–2.76)
	High (N=3)	3.89 (0.86–17.64)		3.43 (0.75–15.66)
Vapor and gases				
	All exposed (N=29)	1.15 (0.73–1.83)		1.09 (0.68–1.73)
	High (N=0)	NA		NA
	Low (N=29)	1.26 (0.80–2.00)		1.18 (0.74–1.90)
Organic dust				
	All exposed (N=24)	1.92 (1.16–3.17)		1.76 (1.05–2.95)
	Low (N=22)	1.78 (1.06–3.01) ^c^		1.63 (0.95–2.78) ^d^
High (N=2)				
Fumes				
	All exposed (N=18)	0.77 (0.44–1.36)		0.71 (0.40–1.26)
	Low (N=18)	0.83 (0.47–1.45)		0.76 (0.43–1.35)
	High (N=0)	NA		NA
**Women (N=109)**				
Inorganic dust including silica				
	All exposed (N=12)	0.85 (0.44–1.64)		0.87 (0.44–1.70)
	Low (N=12)	0.85 (0.44–1.64)		0.87 (0.44–1.70)
	High (N=0)	NA		N.a
Silica dust				
	All exposed (N=2)	2.24 (0.40–12.61)		2.23 (0.40–12.57)
	Low (N=2)	2.24 (0.40–12.62)		2.23 (0.40–12.57)
	High (N=0)	NA		NA
Vapor and gases				
	All exposed (N=14)	0.64 (0.35–1.17)		0.65 (0.35–1.19)
	Low (N=14)	0.64 (0.36–1.18)		0.65 (0.36–1.19)
	High (N=0)	N.a		NA
Organic dust				
	All exposed (N=1)	0.21 (0.03–1.62)		0.21 (0.03–1.62)
	Low (N=1)	0.23 (0.03–1.26)		0.22 (0.03–1.78)
	High (N=0)	NA		NA
Fumes				
	All exposed (N=17)	0.73 (0.42–1.29)		0.73 (0.42–1.26)
	Low (N=17)	0.73 (0.42–1.26)		0.74 (0.42–1.29)
	High (N=0)	NA		NA

^a^ Matched for age. ^b^ Adjusted for educational level (N=102 men). ^c^ P for trend = 0.006. ^d^ P for trend = 0.02.

Because the JEM-classified exposures are overlapping, we analyzed all exposures in single multivariable models (tables 4 and 5). In the model not separating silica from other inorganic dust, there was an increased odds for organic dust among men 1.86 (95% CI 1.10–3.12), with slightly lower OR (95% CI 1.77, 1.04–3.00) when adjusting for educational level (table 4). There were no increased OR among women. When separating between silica dust and inorganic dust, exposure to organic dust showed increased odds among men [OR 1.83 (95% CI 1.08–3.08)] with lower odds after adjusting for educational level [OR 1.68 (95% CI 0.98–2.87)]. For silica dust in the same model, the OR was 1.27 (95% CI 0.54–2.96), whereas the OR for inorganic dust without silica was 0.98 (95% CI 0.57–1.70).

**Table 4:** Logistic regression models of risks of pulmonary alveolar proteinosis (PAP) in relation to occupational exposure to inorganic dust, vapor and gases, fumes, and organic dust with all exposures in the same model. All exposed, low exposed and high exposed are compared with unexposed. [OR=odds ratios; CI=confidence interval].

Occupational exposures		Basic model		Adjusted model ^a^
		OR (95% CI)		OR (95% CI)
All				
	Inorganic dust including silica	1.12 (0.73–1.71)		1.04 (0.68–1.61)
	Vapor and gases	0.96 (0.63–1.47)		0.97 (0.63–1.49)
	Organic dust	1.40 (0.86–2.29)		1.34 (0.81–2.20)
	Fumes	0.75 (0.49–1.15)		0.74 (0.48–1.14)
Men (N=103)				
	Inorganic dust including silica	1.05 (0.64–1.71)		0.97 (0.59–1.60)
	Vapor and gases	1.22 (0.73–2.04)		1.23 (0.74–2.07)
	Organic dust	1.86 (1.10–3.12)		1.77 (1.04–3.00)
	Fumes	0.76 (0.42–1.37)		0.75 (0.41–1.35)
Women (N=109)				
	Inorganic dust including silica	1.62 (0.64–4.09)		1.56 (0.61–3.96)
	Vapor and gases	0.57 (0.24–1.36)		0.58 (0.24–1.38)
	Organic dust	0.18 (0.02–1.53)		0.19 (0.02–1.57)
	Fumes	0.82 (0.42–1.58)		0.81 (0.42–1.57)
^a^ Adjusted for educational level.				

**Table 5:** Logistic regression models of risks of pulmonary alveolar proteinosis (PAP) in relation to occupational exposure to inorganic dust excluding silica, silica dust only, vapor and gases, fumes, and organic dust with all exposures in the same model. All exposed, low exposed and high exposed are compared with unexposed. [OR=odds ratios; 95% CI=95% confidence interval].

Occupational exposures		Basic model		Adjusted model ^a^
		OR (95% CI)		OR (95% CI)
All (N=212)				
	Inorganic dust excluding silica	1.04 (0.66–1.65)		0.95 (0.59–1.53)
	Silica dust only	1.48 (0.69–3.18)		1.44 (0.67–3.11)
	Vapor and gases	0.99 (0.64–1.52)		1.00 (0.65–1.55)
	Organic dust	1.37 (0.83–2.24)		1.30 (0.79–2.14)
	Fumes	0.75 (0.49–1.15)		0.73 (0.48–1.13)
Men (N=103)				
	Inorganic dust excluding silica	0.98 (0.57–1.70)		0.85 (0.49–1.50)
	Silica dust only	1.27 (0.54–2.96)		1.21 (0.52–2.82)
	Vapor and gases	1.26 (0.74–2.14)		1.27 (0.76–2.16)
	Organic dust	1.83 (1.08–3.08)		1.68 (0.98–2.87)
	Fumes	0.75 (0.41–1.35)		0.71 (0.39–1.28)

^a^ Adjusted for educational level.

We also analyzed the effect of cumulative exposure of a maximum of five years before the index date, restricted to the population with occupational data before index date (table 6). There was a statistically significant association by duration: the longer exposure to inorganic dust including silica was associated with PAP, regression coefficient 0.089 (P=0.049) among all, as well as amongst the men 0.12 (P=0.03). There also was a positive association for organic dust among men, 0.16 (P=0.02). For fumes, among all there was a negative association with cumulative years of exposure of borderline statistical significance, -0.11 (P=0.05). There were no statistically significant associations for women, and the direction of association was positive only for inorganic dust and silica alone (table 6).

**Table 6:** Regression models of estimates for risks of pulmonary alveolar proteinosis (PAP) in relation to occupational exposure to inorganic dust, silica dust, vapor and gases, fumes, and organic dust compared with the unexposed. The estimates are based on yearly exposure, cumulative over five years (N=148 cases). [SE=standard error]

Occupational exposure		Regression coefficient (SE)	P-value
Inorganic dust including silica			
	All	0.089 (0.045)	0.049
	Men	0.12 (0.055)	0.03
	Women	0.029 (0.08)	0.72
Silica dust			
	All	0.067 (0.095)	0.48
	Men	0.43 (0.10)	0.68
	Women	0.22 (0.24)	0.36
Vapor and gases			
	All	0.021 (0.047)	0.65
	Men	0.036 (0.059)	0.54
	Women	-0.11 (0.08)	0.17
Organic dust			
	All	0.08 (0.064)	0.18
	Men	0.16 (0.068)	0.02
	Women	-0.67 (0.50)	0.19
Fumes			
	All	-0.11 (0.054)	0.05
	Men	-0.11 (0.075)	0.14
	Women	-0.10 (0.080)	0.19

Of the 24 men with PAP who were exposed to organic dust, the distribution was as follows: 6 worked as carpenters, 3 as building/frame workers, 1 as a logger and 1 as wood-processing operator; 9 worked with livestock either as farmers (N=2) or as animal producers (N=7); 2 worked in a bakery, 1 was a paper mill worker and 1 a librarian (thus presumably with low exposure to paper dust).

## Discussion

This population-based registry-utilizing case-control study indicates that occupational exposure to organic dust among men may contribute to PAP risk. These findings extend previous observations based on case reports indicating that PAP is more common among occupations traditionally characterized as “dusty trades”.

Amongst men, the exposure to organic dust showed an estimated doubling of the odds for PAP, with slightly lower risk in models adjusted for educational attainment. This was not seen amongst women, which accounts for a lowered OR when we analyzed men and women combined. The 24 men with PAP exposed to organic dust had occupations implying exposure to agricultural dust (N=11), wood dust (N=11), or paper dust (N=2) (including the librarian).

We also analyzed the effect of cumulative exposure as a linear trend of the likelihood of PAP by year up to five years of exposure. This lends further support to an association among men between exposure to organic dust and PAP among men, but it also reinforced the pattern we observed of negative or no association with exposure among women. Further, there also was a linear trend for exposure to inorganic dust and PAP among men. We were limited in our ability to analyze cumulative exposure over a longer period than five years due to small numbers with exposure dating back in time beyond that. However, it is notable that case reports of occupational PAP suggest a relatively short latency between exposure and disease. These include sandblasters exposed to silica for fewer than four years prior to symptom-onset and indium-tin oxide manufacturing workers who developed symptoms within nine months of first exposure (16, 17). Our utilization of data on occupational exposures during the one year and five years before disease onset is consistent with these clinical observations.

Multiple case series of PAP have implicated increased prevalence of occupations with exposure to dust, both organic and inorganic (11, 12). It should be stressed, however, that these case studies do not include referents for comparison. Nonetheless, dusty trades seem to be over-represented in the reported PAP case series, both those involving organic and inorganic dust. As noted previously, the initial publication on PAP dating from 1958 described 27 cases of whom 9 had occupational exposure to organic dust and 3 to silica (1). Occupations with exposure to wood dust specifically are prominent in several PAP case series (18–20). Hence, we conclude that our results indicate that occupational exposure to organic dust is a risk factor for PAP.

We could not confirm an association between PAP and exposure to silica dust, even if the risk estimates were greater than one. Of note, the established entity of “acute silico-proteinosis” well recognized since the 1930s, is in retrospect is indistinguishable pathologically from PAP (21–23). This condition, which could be framed “silica-related PAP” occurs after weeks to months with relative high exposure to silica dust (12, 24). In our study, the indications of increased risk for PAP in relation to silica exposure were observed amongst the high exposed, although there were few cases (N=3) and inconclusive results.

There is ample biological plausibility for the role of dusty trades in causing PAP. The inhalation of dust is suggested to stimulate increased surfactant production from Type II cells. The alveolar macrophages, responsible for surfactant clearance gradually become overloaded, resulting in an intracellular accumulation of lipids and foamy macrophages, a hallmark of PAP. Exposure to pine dust and agricultural dust also adversely affects alveolar macrophages (25, 26).

However, there is a limitation in the present study in that we were not able to separate the cases into the different subtypes: autoimmune, secondary, and congenital PAP. This information was lacking in the national registries. As we only include cases with adult-onset PAP, our cases probably include only autoimmune and secondary PAP. Of all PAP cases, the autoimmune subtype constitutes 90% (27). This implies that most of our cases were of the autoimmune subtype.

The cases are included based on their first contact with healthcare for 1991–2022 (a period of 32 years) in the age interval 20–65 years. This means that the cases are a mixture of prevalent and incident events, especially in the beginning of the period, as we then catch prevalent cases with onset before 1991. Potential cases with onset before 1991 may have died, which lead to a survival bias, ie, there is likely an underrepresentation of cases with more severe disease in the earlier period. Another limitation may be systematic misclassification error in the exposure identification for cases included as prevalent cases if we do not correctly identify the exposure before the onset of PAP. For example, a case that is still able to work but, due to symptoms of PAP, must change job to one with lower exposure.

In the present study, we also present models where we have adjusted for educational level as a marker of socioeconomic status. Adjustments for socioeconomic status can be controversial in occupational epidemiology. If educational level is a predictor of both exposure and PAP, that would be a rationale for such adjustment. On the other hand, if educational level is related to occupational exposures upstream in the causal pathway (for example, because those with lower education take on dustier jobs) that could be a source of overadjustment and potentially systematically bias the exposure estimates toward the null. For that reason, although we do present the results of an educationally adjusted model, we consider the estimates without educational adjustments to be more reliable.

Another limitation is that the occupational exposures we analyzed were based on registry data (eg, occupational titles) rather than by self-report. Further, these also were transformed further to capture exposure likelihood through applying an established JEM. The JEM was based on Swedish exposure assessments from the 1980s and 1990s, and several experienced occupational hygienists were involved in its development (14). The application of a JEM is a systematic way of transforming occupational titles and branches into occupational exposures (28). One important potential limitation of using JEM is their lower sensitivity compared to other methods, such as self-assessed exposures (28, 29). Supporting the use of JEM, however, is the potential for recall bias using self-assessed exposures in case–control studies (28). Exposure assignment based on the JEM approach can create an exposure misclassification that can be a non-differential misclassification. This introduces an error of Berkson type that will not bias the risk estimates but will lead to a reduction power and wider CI (28, 30).

We only analyzed occupational exposure based on employment years before the disease onset or in a limited number soon after disease onset. Occupation the year before index date was used as a proxy for a longer duration of exposure. In addition, among the cases where we had data about the occupations five years before index date, we also analyzed the effect of five years of cumulative exposure. These analyzes indicated a positive exposure–effect relation between organic and inorganic dust and risk of PAP, further supporting our main findings.

As another limitation, it also is important to stress that we did not have cigarette smoking data for either the cases or the controls. In Sweden, the prevalence of current smoking among those aged 50–65 years is approximately 17% (31, 32). As noted previously, current smoking may be a risk factor in PAP, at least for more severe disease. Therefore, we cannot exclude current smoking as a potential, unaccounted for confounder. When we adjusted for educational attainment, which is known to be linked to smoking status in Sweden, risk was attenuated. But this attenuation also is consistent with the interrelationships among social class, education, and poorer working conditions.

Although a major strength of our study is its reliance on national registry data to identify PAP cases, this remains a very rare disease. Thus, 200 cases limit study power. It may very well be that multinational pooled registry data might have provided more robust risk estimates. In general, Swedish patient registers have high quality such that misclassification of disease is unlikely (33).

Because we obtained randomly selected controls from the same national population, we were able to match on key covariates and control for selected potential confounders. Although we did not include any co-morbid conditions as confounders, it is unlikely that common co-morbidities are relevant. In one German study, hypertension and depression were the most common PAP associated co-morbidities (5).

In conclusion, although our findings were suggestive, we could not confirm the relationships reported in case series indicating an association between occupational exposure to inorganic dust in general, and silica dust in particular, and elevated risk of PAP. We did observe a statistically significant association between occupational exposure to organic dust and risk of PAP, but this was present only among men. Despite these limitations our findings, derived from national population-based epidemiologic analysis indicate a potential link between selected occupational exposures and PAP.
